# Supplementary material for: Loss of atm in Zebrafish as a Model of Ataxia–Telangiectasia Syndrome
Source: Biomedicines. 2022 Feb 3;10(2):392. doi: 10.3390/biomedicines10020392 (PMC8962326; doi:10.3390/biomedicines10020392)
Supplement: Supplementary file 1 [file biomedicines-10-00392-s001.zip › biomedicines-1510096-supplementary.pdf]

Supplementary Figure S1

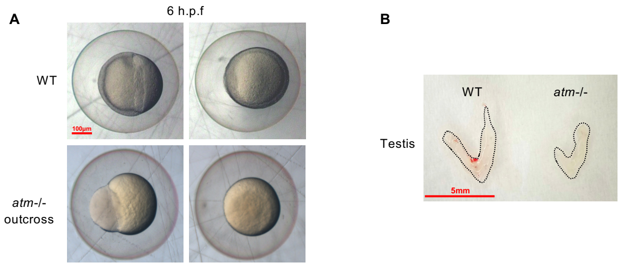

Supplementary Figure S1. Observation of infertility phenotype in *atm-/-* zebrafish. (**A**) 6-hpf embryos from *atm-/-* male fish outcrosses. hpf: hours post fertilization. (**B**) General observation *atm-/-* and WT fish's testis.

Supplementary Figure S2

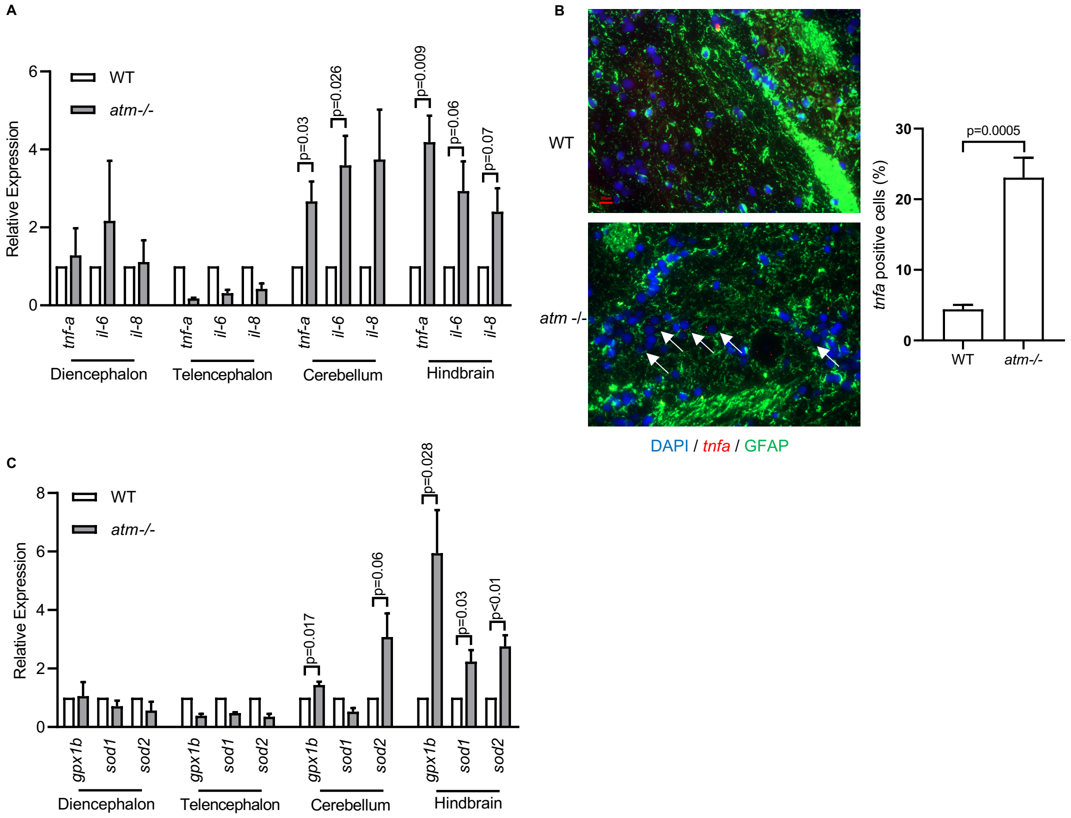

Supplementary Figure S2. Oxidative stress and neural inflammatory in *atm-/-* fish's brain.

(**A**) Relative expression levels of *il -6, il -8* and *tnf-α* genes in diencephalon, telencephalon, cerebellum, and hindbrain of WT and *atm-/-* fish (n=3 for each group). (**B**) Co-detection RNA-scope images of *tnfa* mRNA (red) and GFAP (green) in WT and *atm-/-* fish's hindbrain. Quantification of *tnfa* positive cells in WT and *atm-/-* group (n=5 for WT, n=9 for *atm-/-*). (**C**) Relative expression levels of *gpx-1b, sod1* and *sod2* genes in diencephalon, telencephalon, cerebellum, and hindbrain of WT and *atm-/-* fish (n=3 for each group). The statistical significance is analyzed using the two-tailed Student's t-test. Data are shown in means ± SEM.

Supplementary Figure S3

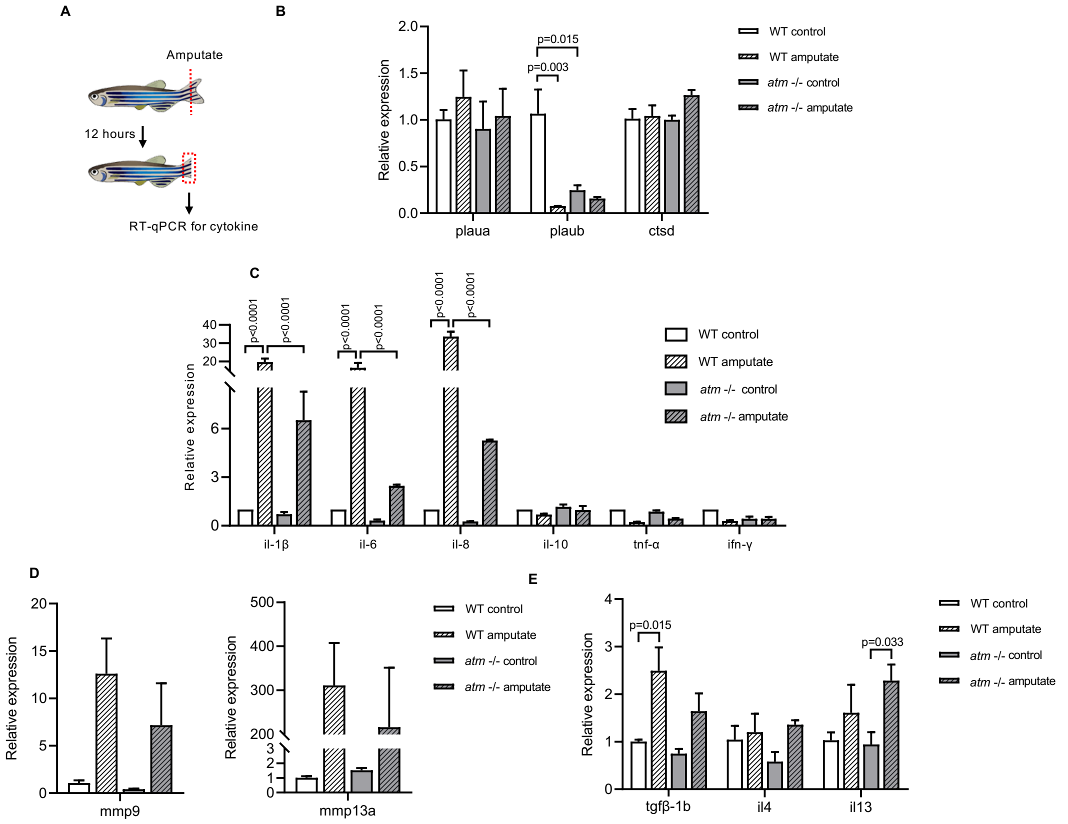

Supplementary Figure S3. The cytokine profile determined 12 hours after caudal fin amputation of *atm-/-* fish.

(**A**) Schematic representation of the caudal fin amputation system used throughout the study. (**B**) Relative expression levels of *plaua, plaub* and *ctsd* genes in WT and *atm-/-* with or without amputation. (**C**) Relative expression levels of *il-1β, il -6, il -8, il -10, tnf-α* and *ifn-γ* genes in WT and *atm-/-* with or without amputation. (**D**) Relative expression levels of *mmp9* and *mmp13a* genes in WT and *atm-/-* with or without amputation. (**E**) Relative expression levels of *tgfβ-1b, il-4* and *il-13* genes in WT and *atm-/-* with or without amputation. The statistical significance is analyzed using one-way ANOVA. n=3 for each group. Data are shown in means ± SEM.

Supplementary Figure S4

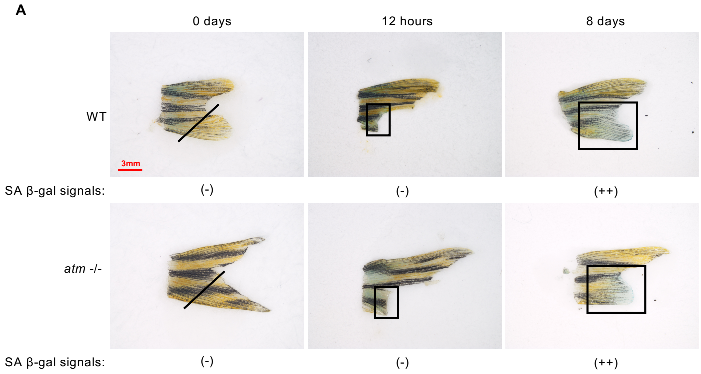

Supplementary Figure S4. Senescence-associated β-galactosidase of amputated caudal fin.

(**A**) Senescence-associated β-galactosidase experiments are operated on amputated caudal fin from WT and *atm*-/- zebrafish after 12 hours and 8 days post-amputation. The non-amputated group served as negative control. The signal levels are identified (n=3 for each group).

Supplementary Figure S5

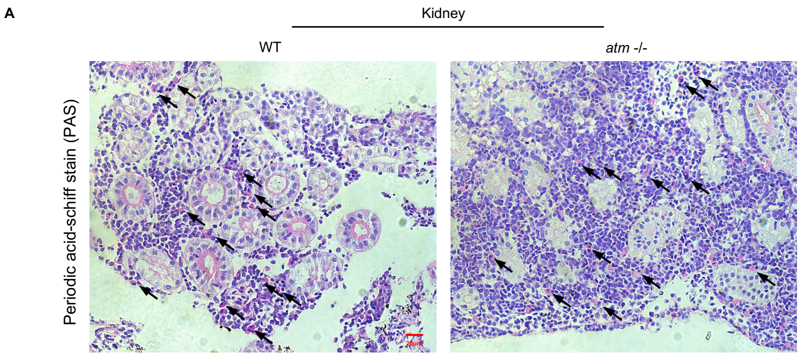

Supplementary Figure S5. Periodic acid-schiff stain of kidney tumor

(**A**) Periodic acid-schiff stain of kidney tumor in *atm-/-* fish compare to WT. Arrows point to the positive signals (n=3 independent experiments).
